# Supplementary material for: Impact of maternal HIV infection on the gut microbiome and metabolome of mothers and infants: the PRACHITi cohort in Pune, India
Source: Nat Commun. 2026 Feb 23;17:3097. doi: 10.1038/s41467-026-69912-0 (PMC13039106; doi:10.1038/s41467-026-69912-0)
Supplement: Supplementary file 1 — Supplementary Information [file 41467_2026_69912_MOESM1_ESM.pdf]

Title: Impact of maternal HIV infection on the gut microbiome and metabolome of mothers and infants: the PRACHITi cohort in Pune, India

Supplementary Materials – Online Only

Methods:

Gut Microbiota

A dual-barcode system and a mapping file linking barcodes to samples were employed, along with the QIIME script (split_libraries.py)[1], to de-multiplex the sequences. The forward and reverse fastq files were then separated by sample using the QIIME script (split_sequence_file_on_sample_ids.py). Primer sequences were removed with TagCleaner version 0.16[2], and data processing followed the DADA2 workflow for large datasets using dada2 v1.5.2 (https://benjjneb.github.io/dada2/bigdata.html)[3]. Forward reads were trimmed to 255 base pairs (bp) and reverse reads to 225 bp, with filtering to exclude ambiguous bases, maintain a minimum quality score of 2, and allow for fewer than two errors based on quality scores. Reads were subsequently merged, and chimeric sequences were removed following the DADA2 protocol.

Metabolomics

Samples were accessioned into Metabolon's Laboratory Information Management System (LIMS), assigned unique identifiers, and stored at −80°C until analysis. Metabolon's proprietary software was used for peak detection, compound identification, and quality control. Metabolites were identified by comparison to a library of over 3,300 authenticated standards based on retention index, accurate mass (±10 ppm), and MS/MS spectral matching. Quality assurance included pooled matrix samples, process blanks, and internal standards spiked into each sample to monitor instrument performance and chromatographic consistency. Samples were randomized across the platform, with QC samples interspersed throughout the run to assess instrument and process variability, calculated using the median relative standard deviation of internal and endogenous metabolites.

Results:

Exploratory analysis by CHEU status at 6 months of age, stratified by feeding type

Additionally, we conducted an exploratory analysis on the association between CHEU and CHUU groups and their gut microbiota profile at 6 months by conducting separate stratified analysis by feeding type. In exclusively breastfed infants, there were no significant differences in α- diversity (**Supplementary Figure 5a**) or β-diversity for model 1 (p=0.234). Similarly, for infants receiving anything other than human milk, neither α-diversity (**Supplementary Figure 5b**) nor β-diversity (p=0.334) differed significantly by infant HIV exposure group.

At the taxa abundance level, stratified by feeding type, we found there were no significant differences among exclusively breastfed infants by HIV exposure status. Among infants that were not exclusively breastfeeding, there were also no significantly different

bacteria based on adjusted p-values, but *Alloprevotella* (lower abundance in CHEU) and *Hungatella* (higher abundance in CHEU) had significant unadjusted p-values (**Supplementary Figure 6**).

Exploratory analyses including non-pregnant women

We also compared the microbiota profile of non-pregnant WHIV and SN. The overall microbiota based on β-diversity ($p<0.001$), but not α diversity, was different by HIV status (**Supplementary Figure 7 and Supplementary Table 5**). Among non-pregnant WHIV, there were 4 significant bacteria all with lower abundance, including *Coprococcus_2, Ruminococcaceae_UCG-014, Christensenellaceae_R-7_group, and Family_XIII_UCG-001,* compared to non-pregnant SN women (**Supplementary Figure 8 and Supplementary Table 12**). A low abundance of *Christensenellaceae_R-7_group* has been found to suppress inflammatory responses[4] and reduced *Coproccocus_2* plays a role in gut inflammation[5]. We also conducted an exploratory analysis to compare the microbiota in pregnant WHIV during the third trimester as compared to SN but did not observe any differences in diversity or taxa abundance analyses.

Supplementary Table 1. Sociodemographic and clinical features of study participants by HIV status at second trimester (N=177)

Characteristic	N (%)[a]			
	Overall (N = 177)	Women with HIV (n = 65 [37%])	Women Seronegative for HIV (n = 112 [63%])	*P*-value[e]
Age, median (IQR)[b]	23 (21-27)	24 (21-28)	23 (20-26)	*0.048*[f]
Anemia				
Yes	75 (43)	29 (45)	46 (41)	0.63
No	100 (57)	35 (55)	65 (59)	
Missing	2	1	1	
Education				
None to High School	158 (89)	59 (91)	99 (88)	0.80
Post High School to Postgraduate	19 (11)	6 (9)	13 (12)	
Smoking Status[d]				
Yes	23 (13)	4 (6)	19 (17)	0.06
No	154 (87)	61 (94)	93 (83)	
Undernutrition (MUAC)[c]				
Yes (< 23 cm)	65 (37)	28 (43)	37 (33)	0.20
No (≥ 23 cm)	111 (63)	37 (57)	74 (67)	
Missing	1	0	1	
Gestational Diabetes				
Yes	15 (9)	7 (11)	8 (7)	0.41
No	153 (91)	54 (89)	99 (93)	
Missing	9	4	5	
CD4 Count, median (IQR)	--	497 (368.25-647.25)	--	NA
ART Regimen				
AZT/3TC/ATV	--	1 (1)	0 (0)	NA
AZT/3TC/NVP	--	7 (11)	0 (0)	

TDF/3TC/EFV	--	46 (72)	0 (0)	
Other[e]	--	10 (16)	0 (0)	
Not Applicable (HIV-)	--	0	112	
HIV Viral Load (VL)				
Undetectable VL (≤ 40 copies/mL)	--	39 (60)	--	NA
Detectable VL (> 40 copies/mL)	--	26 (40)	--	

[a]N is number of individuals; [b]IQR stands for Interquartile Range; [c]Undernutrition is based on Mid-upper arm circumference (MUAC) measurements; [d]Past active smoking status of women. [e]P-values were calculated using a two-sided Fisher exact test for categorical variables and a two-sided Wilcoxon rank sum test for continuous variables to determine the difference in characteristics by HIV status but does not account for differences in the missing category. [e]Other ART regimens include: TDF/3TC/LPV/r, TDF/3TC/ATV/r, TDF/3TC/RAL, and ABC/3TC/NVP. [f]P-values <0.05 are significant and italicized.

Supplementary Table 2. Sociodemographic and clinical features of infants 6 months of age by HIV exposure status (N=177)

Characteristic	N (%)[a] Overall (N = 177)	CHEU[e] (n = 61 [34%])	CHUU[f] (n = 116 [66%])	*P*-value[d]
Maternal Age, median (IQR)[b]	24 (21-27)	25 (21-28)	23 (21-26)	*0.048*[g]
Currently Breastfeeding				*<0.001*
Yes	151 (86)	40 (66)	111 (97)	
No	25 (14)	21 (34)	4 (3)	
Missing	1	0	1	
Maternal Education				0.81
None to High School	156 (88)	53 (87)	98 (84)	
Post High School to Postgraduate	21 (12)	8 (13)	18 (16)	
Maternal Smoking Status[c]				*0.01*
Yes	20 (11)	2 (3)	18 (16)	
No	157 (89)	59 (97)	98 (84)	
Infant's BMI z-score at 6 months				0.09
< -2 z-score	37 (21)	19 (31)	18 (16)	
≥ -2 z-score	136 (79)	42 (69)	94 (84)	
Missing	4	0	4	
Maternal Gestational Diabetes				0.59
Yes	17 (10)	7 (12)	10 (9)	
No	157 (90)	52 (88)	105 (91)	
Missing	3	2	1	
Exclusively Breastfeeding				0.74
Yes, exclusively breastfeeding	60 (34)	22 (36)	38 (33)	

No, not exclusively breastfeeding	116 (66)	39 (64)	77 (67)
Missing	1	0	1

[a]N is number of individuals; [b]IQR stands for Interquartile Range; [c]Past active smoking status of women. [d]P-values were calculated using a two-sided Fisher exact test for categorical variables and a two-sided Wilcoxon rank sum test for continuous variables to determine the difference in characteristics by HIV status but does not account for differences in the missing category. [e]CHEU stands for children who are HIV-exposed uninfected. [f]CHUU stands for children who HIV-unexposed uninfected. [g]P-values <0.05 are significant and italicized.

Supplementary Table 3. Cross-tabulation of maternal HIV status at the third trimester by number of sexual partners in lifetime (N=207)

	WHIV[c] (n=75)	SN[c] (n=132)
1 sexual partner	26 (96%)[b]	18 (100%)
2 or more sexual partners	1 (4%)	0 (0%)
Missing[a]	48	113

[a]Several participants are missing this data as this question was only asked for participants co-enrolled in a sub-study.
[b]Percentages only include participants with data.
[c]WHIV indicates women with HIV; SN indicates women seronegative for HIV.

Supplementary Table 4. Gut microbial average α-diversity similarity by HIV status among a subset of pregnant women with second trimester and third trimester samples using a paired t-test (N=68)

**Index**	**All samples[d] (N=68)**			**WHIV[a] Samples (N=33)[b]**			**SN[a] Samples (N=35)**		
	2T Mean	3T Mean	p-value[c]	2T Mean	3T Mean	p-value	2T Mean	3T Mean	p-value
**Shannon[e]**	2.57	2.56	0.60	2.49	2.52	0.66	2.64	2.59	0.21

[a]WHIV indicates women with HIV; SN indicates women seronegative for HIV; 2T: Second trimester; 3T: Third trimester
[b]N=3 WHIV samples did not have third trimester samples.
[c]p-values are based on a two-sided paired t-test of mean of longitudinal second trimester and mean of longitudinal third trimester samples.
[d]A total of 474 samples were taken every two weeks between second trimester and third trimester.
[e]The Shannon α-diversity index was used to calculate the α-diversity sample means.

Supplementary Table 5. Gut microbial β-diversity (based on Bray-Curtis distance) using PERMANOVA[a] by HIV status (N=207)

Models	Third trimester (N=207)	Second Trimester (N=177)	Postpartum (N=182)	Infants (N=179)	Non-pregnant (N=173)
**Model 0 p-value[b]**	*0.004*[d]	0.05	*0.01*	0.36	*<0.001*[e]
**Model 1 p-value[c]**	*0.004*	0.05	*0.009*	0.35	*<0.001*[f]

[a]PERMANOVA stands for permutational multivariate analysis of variance. This is a two-sided univariable and multivariable test.
[b]Univariable model 0 is the crude model with no adjustments.
[c]Multivariable model 1 adjusted for age, education, and mid-upper arm circumference (MUAC).
[d]Italicized p-values indicate significant difference in β-diversity.
[e]The exact p-value for model 0 for non-pregnant is 0.0002.
[f]The exact p-value for model 1 for non-pregnant is 0.0003.

Supplementary Table 6. Gut microbial α-diversity of all samples from the second and third trimester (including frequent follow-up) by HIV status (N=242)

**Model 1**[a]	**2T + 3T (N=242)**[b]
**Shannon coefficient**	-0.059
**p-value**	0.12

[a]A two-sided linear mixed model with subject as random effect, α-diversity as outcome and HIV status as exposure variable, and adjusting for age, education, and mid-upper arm circumference (MUAC) for model 1.
[b]For 2T (second trimester) + 3T (third trimester), there were 747 samples from 242 individuals.

Supplementary Figure 1. Gut microbial α-diversity boxplots over 3 timepoints by HIV status

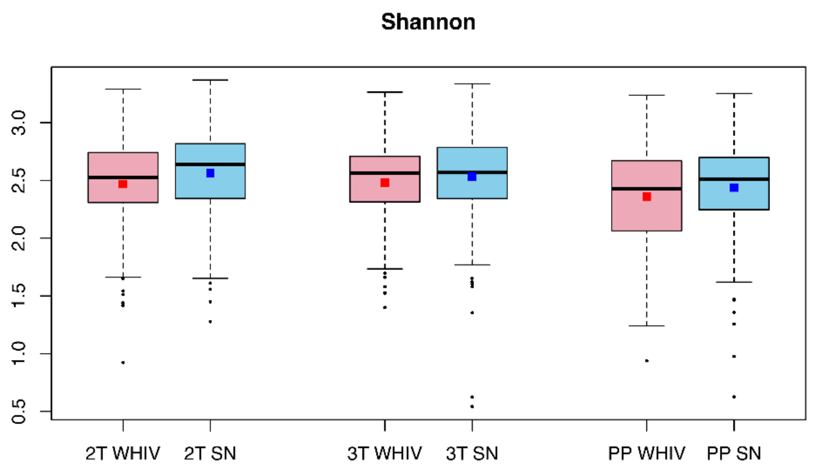

Supplementary Figure 1 Legend. Comparison of α-diversities between WHIV and SN at three different stages of pregnancy: 2nd trimester, 3rd trimester, and 6 months postpartum. WHIV indicates women with HIV. SN indicates women seronegative for HIV. The thick black line represents the median, the colored dot is the mean, the box shows the interquartile range (IQR) with the bottom line as the 25th percentile and top line as the 75th percentile, the lines extending from the box are the whiskers (1.5 x IQR), and dots after the whiskers are outliers. There were 183 individuals (384 longitudinal samples) for second trimester, 210 individuals (363 longitudinal samples) for third trimester, and 182 individuals (182 cross-sectional sample) for postpartum. 2T stands for second trimester, 3T stands for third trimester, and PP stands for postpartum.

Supplementary Table 7. Differential abundance of taxa of all samples from the second and third trimester by HIV status (N=242)[a]

	Model 0		Model 1[b]	
Taxon[e]	LFC[c] [CI][d]	P-value[c]	LFC [CI]	P-value
Megamonas	2.10 [1.05, 3.15]	*0.006*	2.05 [0.98, 3.12][f]	*0.008*
Lachnoclostridium	1.57 [0.89, 2.25]	*0.001*	1.62 [0.93, 2.31]	*0.0009*
Lachnospiraceae_NK4A136_group	-1.29 [-1.98, -0.60]	*0.011*	-1.41 [-2.11, -0.71]	*0.006*
Fusobacterium	1.32 [0.59, 2.06]	*0.014*	1.36 [0.61, 2.11]	*0.013*

[a]There were 747 samples from 242 individuals.

[b]Multivariable model 1 adjusted for subject (random effect), age, middle upper arm circumference (MUAC), and education.

[c]Benjamini-Hochberg adjusted p-values

[d]The Bonferroni adjusted log fold-change (LFC) represents the logarithmic change in corrected absolute abundance of bacteria in the specified model (e.g. higher or lower) in pregnant WHIV compared to SN. If the estimate is positive, higher abundance in WHIV and if negative, lower in WHIV.

[e]The Bonferroni adjusted 95% confidence interval indicates a range of values, upper and lower bound, within which the true LFC lies for each taxa.

[f]As an example of interpretation, the log fold change of *Megamonas* in adjusted model 1 is 2.05 higher in pregnant WHIV compared to those SN during the 2nd and 3rd trimester combined, with a 95% confidence interval of [0.98, 3.12], indicating a statistically significant increase in abundance based on the p-value of 0.008, which is < 0.05. ANCOM-BC analysis methods were used to find these results which is a two-sided test.

Supplementary Table 8. Differentially present or absent taxa identified by ANCOM-BC[a] during pregnancy (N=242)

Taxon	Structural Zero (WHIV)[b]	Structural Zero (SN)[c]
Aggregatibacter	Yes	No
Anaerofilum	Yes	No
Anaerovibrio	Yes	No
Bergeyella	Yes	No
Brachyspira	Yes	No
Candidatus_Methanomethylophilus	Yes	No
Defluviitaleaceae_UCG-011	Yes	No
Epulopiscium	Yes	No
Erysipelotrichaceae_UCG-006	Yes	No
Lachnoanaerobaculum	Yes	No
Lachnospiraceae_UCG-003	Yes	No
Morganella	Yes	No
Prevotellaceae_UCG-003	Yes	No
Prevotellaceae_UCG-004	Yes	No
Shimwellia	Yes	No
Treponema_2	Yes	No
Aeromonas	No	Yes
Aliihoeflea	No	Yes
Clostridioides	No	Yes

DTU089	No	Yes
Enhydrobacter	No	Yes
Faecalicoccus	No	Yes
Faecalitalea	No	Yes
Mycoplasma	No	Yes
Pediococcus	No	Yes
Pseudomonas	No	Yes
Pyramidobacter	No	Yes
S5-A14a	No	Yes

[a]ANCOM-BC stands for Analysis of Compositions of Microbiomes with Bias Correction method and we used multivariable model 1 which adjusted for age, middle upper arm circumference (MUAC), and education.

[b]WHIV stands for women who have HIV. 'Yes' for structural zero for WHIV and 'No' in SN means the bacteria is differentially absent (i.e. absent in WHIV but present in SN).

[c]SN stands for seronegative HIV. 'No' for structural zero for WHIV and 'Yes' in SN means the bacteria is differentially present (i.e. present in WHIV but absent in SN).

<u>Supplementary Table 9.</u> Significant metabolites in the metabolomics at third trimester by HIV status (N=100)[a]

Metabolite	Super Pathway	Sub Pathway	mean WHIV	mean SN	mean WHIV - mean SN	Test Statistic	Raw p-value	BH adjusted p-value[b]	Group Significance[c]
5alpha-pregnan-3beta,20alpha-diol disulfate	Lipid	Progestin Steroids	-0.50	0.31	-0.81	-4.86	0.0000012	0.0005	WHIV < SN
5alpha-pregnan-3beta,20alpha-diol monosulfate (2)	Lipid	Progestin Steroids	-0.59	0.30	-0.89	-4.84	0.0000013	0.0005	WHIV < SN
5alpha-pregnan-3beta-ol,20-one sulfate	Lipid	Progestin Steroids	-0.74	0.65	-1.39	-4.80	0.0000016	0.0005	WHIV < SN
5alpha-pregnan-3beta,20alpha-diol monosulfate (1)	Lipid	Progestin Steroids	-1.14	0.39	-1.53	-4.73	0.0000022	0.0005	WHIV < SN
hydroxypalmitoyl sphingomyelin (d18:1/16:0(OH))**	Lipid	Sphingomyelins	-0.27	0.15	-0.42	-4.29	0.000018	0.0017	WHIV < SN
glycocholenate sulfate*	Lipid	Secondary Bile Acid Metabolism	-0.31	0.22	-0.52	-4.10	0.000041	0.0033	WHIV < SN
glycoursodeoxycholate	Lipid	Secondary Bile Acid Metabolism	-0.89	0.24	-1.13	-4.08	0.000045	0.0033	WHIV < SN
sphingomyelin (d18:2/23:0, d18:1/23:1, d17:1/24:1)*	Lipid	Sphingomyelins	-0.18	0.10	-0.29	-3.87	0.00011	0.0062	WHIV < SN
palmitoyl-sphingosine-phosphoethanolamine (d18:1/16:0)	Lipid	Ceramide PEs	-0.29	0.07	-0.36	-3.86	0.00011	0.0062	WHIV < SN
behenoyl dihydrosphingomyelin (d18:0/22:0)*	Lipid	Dihydrosphingomyelins	-0.31	0.15	-0.45	-3.80	0.00015	0.0068	WHIV < SN
tricosanoyl sphingomyelin (d18:1/23:0)*	Lipid	Sphingomyelins	-0.19	0.10	-0.28	-3.79	0.00015	0.0068	WHIV < SN

glycosyl ceramide (d18:2/24:1, d18:1/24:2)*	Lipid	Hexosylceramides (HCER)	-0.31	0.09	-0.40	-3.79	0.00015	0.0068	WHIV < SN
5alpha-androstan-3beta,17beta-diol disulfate	Lipid	Androgenic Steroids	-0.65	0.24	-0.88	-3.78	0.00016	0.0068	WHIV < SN
sphingomyelin (d18:0/20:0, d16:0/22:0)*	Lipid	Dihydrosphingomyelins	-0.31	0.16	-0.47	-3.77	0.00016	0.0068	WHIV < SN
21-hydroxypregnanolone disulfate	Lipid	Pregnenolone Steroids	-0.34	0.41	-0.75	-3.76	0.00017	0.0068	WHIV < SN
pregnanolone/allopregnanolone sulfate	Lipid	Progestin Steroids	-0.37	0.25	-0.62	-3.68	0.00023	0.0082	WHIV < SN
epiandrosterone sulfate	Lipid	Androgenic Steroids	-1.03	0.10	-1.12	-3.65	0.00026	0.0089	WHIV < SN
glycosyl-N-(2-hydroxynervonoyl)-sphingosine (d18:1/24:1(2OH))*	Lipid	Hexosylceramides (HCER)	-1.39	-0.21	-1.18	-3.51	0.00045	0.013	WHIV < SN
lactosyl-N-palmitoyl-sphingosine (d18:1/16:0)	Lipid	Lactosylceramides (LCER)	-0.21	0.05	-0.27	-3.42	0.00062	0.016	WHIV < SN
sphingomyelin (d18:2/21:0, d16:2/23:0)*	Lipid	Sphingomyelins	-0.17	0.12	-0.28	-3.42	0.00063	0.016	WHIV < SN
palmitoyl dihydrosphingomyelin (d18:0/16:0)*	Lipid	Dihydrosphingomyelins	-0.22	0.09	-0.30	-3.37	0.00076	0.019	WHIV < SN
lactosyl-N-nervonoyl-sphingosine (d18:1/24:1)*	Lipid	Lactosylceramides (LCER)	-0.24	0.13	-0.37	-3.30	0.00095	0.022	WHIV < SN
glycolithocholate sulfate*	Lipid	Secondary Bile Acid Metabolism	-0.55	0.17	-0.72	-3.28	0.0010	0.022	WHIV < SN
glycosyl-N-stearoyl-sphingosine (d18:1/18:0)	Lipid	Hexosylceramides (HCER)	-0.29	0.11	-0.40	-3.26	0.0011	0.024	WHIV < SN

1-(1-enyl-palmitoyl)-2-oleoyl-GPC (P-16:0/18:1)*	Lipid	Plasmalogen	-0.18	0.09	-0.27	-3.25	0.0012	0.024	WHIV < SN
palmitoyl sphingomyelin (d18:1/16:0)	Lipid	Sphingomyelins	-0.13	0.05	-0.18	-3.24	0.0012	0.024	WHIV < SN
sphingomyelin (d18:2/24:2)*	Lipid	Sphingomyelins	-0.18	0.05	-0.22	-3.23	0.0012	0.024	WHIV < SN
glycosyl ceramide (d18:1/20:0, d16:1/22:0)*	Lipid	Hexosylceramides (HCER)	-0.19	0.12	-0.31	-3.16	0.0016	0.028	WHIV < SN
androsterone glucuronide	Lipid	Androgenic Steroids	-1.02	-0.27	-0.75	-3.13	0.0017	0.029	WHIV < SN
sphingomyelin (d18:2/24:1, d18:1/24:2)*	Lipid	Sphingomyelins	-0.16	0.04	-0.21	-3.12	0.0018	0.029	WHIV < SN
pregnenolone sulfate	Lipid	Pregnenolone Steroids	-0.25	0.19	-0.43	-3.12	0.0018	0.029	WHIV < SN
myristoyl dihydrosphingomyelin (d18:0/14:0)*	Lipid	Dihydrosphingomyelins	-0.23	0.08	-0.31	-3.12	0.0018	0.029	WHIV < SN
sphingomyelin (d18:1/22:2, d18:2/22:1, d16:1/24:2)*	Lipid	Sphingomyelins	-0.12	0.08	-0.20	-3.10	0.0019	0.029	WHIV < SN
4-hydroxyglutamate	Amino Acid	Glutamate Metabolism	-0.49	0.14	-0.63	-3.10	0.0020	0.029	WHIV < SN
taurolithocholate 3-sulfate	Lipid	Secondary Bile Acid Metabolism	-0.82	0.02	-0.85	-3.04	0.0023	0.0341	WHIV < SN
lignoceroyl sphingomyelin (d18:1/24:0)	Lipid	Sphingomyelins	-0.15	0.07	-0.22	-3.02	0.0025	0.035	WHIV < SN
sphingomyelin (d18:1/24:1, d18:2/24:0)*	Lipid	Sphingomyelins	-0.18	0.03	-0.21	-3.01	0.0026	0.036	WHIV < SN
argininate*	Amino Acid	Urea cycle; Arginine and Proline Metabolism	-0.20	0.28	-0.48	-3.00	0.0027	0.036	WHIV < SN
citrate	Energy	TCA Cycle	-0.11	0.06	-0.17	-2.93	0.0034	0.040	WHIV < SN

sphingomyelin (d18:1/21:0, d17:1/22:0, d16:1/23:0)*	Lipid	Sphingomyelins	-0.18	0.08	-0.25	-2.91	0.0036	0.041	WHIV < SN
sphingomyelin (d18:2/23:1)*	Lipid	Sphingomyelins	-0.15	0.09	-0.24	-2.91	0.0036	0.041	WHIV < SN
sphingomyelin (d18:2/14:0, d18:1/14:1)*	Lipid	Sphingomyelins	-0.16	0.07	-0.23	-2.90	0.0037	0.041	WHIV < SN
5-methyluridine (ribothymidine)	Nucleotide	Pyrimidine Metabolism, Uracil containing	-0.07	0.04	-0.11	-2.87	0.0041	0.045	WHIV < SN
sphingomyelin (d18:0/18:0, d19:0/17:0)*	Lipid	Dihydrosphingomyelins	-0.20	0.12	-0.32	-2.87	0.0041	0.045	WHIV < SN
glycochenodeoxycholate	Lipid	Primary Bile Acid Metabolism	-0.36	0.20	-0.56	-2.85	0.0043	0.047	WHIV < SN
ursodeoxycholate	Lipid	Secondary Bile Acid Metabolism	-1.06	-0.40	-0.65	-2.84	0.0045	0.047	WHIV < SN
1-(1-enyl-palmitoyl)-2-palmitoyl-GPC (P-16:0/16:0)*	Lipid	Plasmalogen	-0.16	0.08	-0.24	-2.84	0.0046	0.047	WHIV < SN
cytosine	Nucleotide	Pyrimidine Metabolism, Cytidine containing	0.08	-1.23	1.31	4.58	0.0000046	0.0007	WHIV > SN
tetrahydrocortisol glucuronide	Lipid	Corticosteroids	0.14	-0.72	0.86	4.56	0.0000051	0.0007	WHIV > SN
glucuronate	Carbohydrate	Aminosugar Metabolism	0.27	-0.07	0.34	4.54	0.0000055	0.0007	WHIV > SN
methionine sulfone	Amino Acid	Methionine, Cysteine, SAM and Taurine Metabolism	0.48	-0.20	0.68	4.44	0.0000089	0.0010	WHIV > SN
mannonate*	Xenobiotics	Food Component/Plant	0.15	-0.15	0.31	4.27	0.000019	0.0017	WHIV > SN
arabitol/xylitol	Carbohydrate	Pentose Metabolism	0.15	-0.14	0.29	4.06	0.00005	0.0034	WHIV > SN
N-acetyl-2-aminooctanoate*	Lipid	N-acyl amino acids	-0.07	-1.02	0.95	3.88	0.00010	0.0062	WHIV > SN

N4-acetylcytidine	Nucleotide	Pyrimidine Metabolism, Cytidine containing	-0.15	-0.92	0.77	3.73	0.00019	0.0074	WHIV > SN
acisoga	Amino Acid	Polyamine Metabolism	0.26	-0.23	0.49	3.71	0.00020	0.0075	WHIV > SN
N1-methylinosine	Nucleotide	Purine Metabolism, (Hypo)Xanthine/Inosine containing	0.18	-0.09	0.27	3.56	0.00038	0.012	WHIV > SN
N2,N2-dimethylguanosine	Nucleotide	Purine Metabolism, Guanine containing	0.15	-0.06	0.21	3.54	0.00040	0.013	WHIV > SN
N6-carbamoylthreonyladenosine	Nucleotide	Purine Metabolism, Adenine containing	0.11	-0.10	0.22	3.53	0.00042	0.013	WHIV > SN
hexanoylglutamine	Lipid	Fatty Acid Metabolism (Acyl Glutamine)	0.27	-0.65	0.92	3.46	0.00054	0.015	WHIV > SN
suberoylcarnitine (C8-DC)	Lipid	Fatty Acid Metabolism (Acyl Carnitine, Dicarboxylate)	-0.25	-0.94	0.69	3.44	0.00057	0.016	WHIV > SN
N-methylpipecolate	Xenobiotics	Bacterial/Fungal	0.14	-0.72	0.87	3.38	0.00072	0.018	WHIV > SN
tetrahydrocortisone glucuronide (5)	Lipid	Corticosteroids	-0.56	-1.01	0.45	3.35	0.00082	0.019	WHIV > SN
trigonelline (N'-methylnicotinate)	Cofactors and Vitamins	Nicotinate and Nicotinamide Metabolism	0.29	-0.21	0.50	3.30	0.00098	0.022	WHIV > SN
3-aminoisobutyrate	Nucleotide	Pyrimidine Metabolism, Thymine containing	0.22	-0.25	0.47	3.30	0.00098	0.022	WHIV > SN
2-amino-4-cyanobutanoate	Amino Acid	Glutamate Metabolism	0.28	-0.10	0.38	3.22	0.0013	0.024	WHIV > SN
phenylacetylglutamate	Peptide	Acetylated Peptides	-0.52	-1.23	0.70	3.22	0.0013	0.024	WHIV > SN

cis-4-decenoylcarnitine (C10:1)	Lipid	Fatty Acid Metabolism (Acyl Carnitine, Monounsaturated)	0.13	-0.26	0.39	3.21	0.0013	0.0242	WHIV > SN
N-acetylglycine	Amino Acid	Glycine, Serine and Threonine Metabolism	0.19	-0.15	0.33	3.20	0.0014	0.025	WHIV > SN
N-acetyl-S-allyl-cysteine	Xenobiotics	Food Component/Plant	-0.78	-1.31	0.53	3.13	0.0018	0.029	WHIV > SN
3-methoxytyramine sulfate	Amino Acid	Tyrosine Metabolism	-0.07	-0.58	0.51	3.11	0.0018	0.029	WHIV > SN
succinoyltaurine	Amino Acid	Methionine, Cysteine, SAM and Taurine Metabolism	-0.34	-0.86	0.52	3.10	0.0019	0.029	WHIV > SN
mannose	Carbohydrate	Fructose, Mannose and Galactose Metabolism	0.06	-0.08	0.14	3.04	0.0024	0.034	WHIV > SN
3,4-dihydroxybutyrate	Lipid	Fatty Acid, Dihydroxy	0.11	-0.05	0.16	3.03	0.0024	0.034	WHIV > SN
N2-acetyl,N6,N6-dimethyllysine	Amino Acid	Lysine Metabolism	0.25	-0.51	0.76	3.01	0.0026	0.036	WHIV > SN
cis-4-decenoate (10:1n6)*	Lipid	Medium Chain Fatty Acid	0.31	-0.21	0.52	2.98	0.0029	0.038	WHIV > SN
3-hydroxy-3-methylglutarate	Lipid	Mevalonate Metabolism	0.13	-0.06	0.19	2.97	0.0030	0.038	WHIV > SN
2-hydroxy-3-methylvalerate	Amino Acid	Leucine, Isoleucine and Valine Metabolism	0.21	-0.11	0.32	2.97	0.0030	0.039	WHIV > SN
1-stearoyl-2-oleoyl-GPS (18:0/18:1)	Lipid	Phosphatidylserine (PS)	-0.36	-1.14	0.78	2.95	0.0032	0.040	WHIV > SN
vanillactate	Amino Acid	Tyrosine Metabolism	0.16	-0.14	0.30	2.95	0.0032	0.040	WHIV > SN
3-hydroxydecanoylcarnitine	Lipid	Fatty Acid Metabolism (Acyl Carnitine, Hydroxy)	-0.02	-0.70	0.69	2.95	0.0032	0.040	WHIV > SN
2-methylbutyrylcarnitine (C5)	Amino Acid	Leucine, Isoleucine and Valine Metabolism	0.12	-0.35	0.46	2.94	0.0033	0.040	WHIV > SN

linoleoyl-linoleoyl-glycerol (18:2/18:2) [1]*	Lipid	Diacylglycerol	0.09	-0.19	0.28	2.92	0.0035	0.041	WHIV > SN
pimeloylcarnitine/3-methyladipoylcarnitine (C7-DC)	Lipid	Fatty Acid Metabolism (Acyl Carnitine, Dicarboxylate)	-0.23	-0.81	0.58	2.91	0.0037	0.041	WHIV > SN
myristoleoylcarnitine (C14:1)*	Lipid	Fatty Acid Metabolism (Acyl Carnitine, Monounsaturated)	0.17	-0.43	0.61	2.85	0.0044	0.047	WHIV > SN
1-stearoyl-2-arachidonoyl-GPS (18:0/20:4)	Lipid	Phosphatidylserine (PS)	0.12	-0.46	0.58	2.83	0.0047	0.048	WHIV > SN
3-hydroxyadipate	Lipid	Fatty Acid, Dicarboxylate	-0.50	-1.15	0.65	2.82	0.0049	0.049	WHIV > SN

[a]Results are based on a two-sided logistic regression with model 1 which adjusted for age, education, and mid-upper arm circumference (MUAC).
[b]Benjamini-Hochberg adjusted p-value
[c]Comparison between WHIV and SN individuals, with the metabolite being significantly higher in the WHIV group if WHIV > SN, and the metabolite being significantly lower in the SN group if WHIV < SN.

Supplementary Table 10. Multi-omics of third trimester samples between significant taxa and metabolites (N=88)

Genus	Chemical	Correlation[a]	Genus p-value[b]	Genus BH-adjusted p-value[c]	Chemical p-value	Chemical BH-adjusted p-value
Fusobacterium	2-hydroxyglutarate	0.43	0.0020	0.21	0.00098	0.015
Prevotella_2	sphingomyelin (d18:1/24:1, d18:2/24:0)*	0.34	0.0361	0.61	0.00094	0.015
Fusobacterium	phenol glucuronide	0.32	0.0020	0.21	0.0031	0.034
Ruminococcaceae_UCG-005	lithocholate sulfate (1)	0.31	0.0638	0.61	0.0029	0.032
Turicibacter	glycoursodeoxycholate	0.31	0.0788	0.62	0.000003	0.00019
Family_XIII_UCG-001	glycoursodeoxycholate	0.31	0.0684	0.61	0.000003	0.00019
Ruminococcaceae_UCG-003	pregnenolone sulfate	0.31	0.0077	0.40	0.00011	0.0032
Dialister	sphingomyelin (d18:2/23:1)*	0.31	0.0628	0.61	0.0017	0.022
Family_XIII_UCG-001	glycolithocholate sulfate*	0.30	0.0684	0.61	0.0010	0.015
Ruminococcaceae_UCG-003	5alpha-pregnan-3beta,20alpha-diol monosulfate (2)	0.30	0.0077	0.40	<0.0001[d]	<0.0001
Fusobacterium	hydroxypalmitoyl sphingomyelin (d18:1/16:0(OH))**	-0.47	0.0020	0.21	<0.0001[e]	<0.0001
Fusobacterium	palmitoyl-sphingosine-phosphoethanolamine (d18:1/16:0)	-0.44	0.0020	0.21	0.00003	0.0011
Fusobacterium	glycosyl ceramide (d18:1/20:0, d16:1/22:0)*	-0.42	0.0020	0.21	0.00046	0.0085
Fusobacterium	myristoyl dihydrosphingomyelin (d18:0/14:0)*	-0.40	0.0020	0.21	0.0023	0.028

Fusobacterium	sphingomyelin (d18:2/23:1)*	-0.39	0.0020	0.21	0.0017	0.022
Fusobacterium	palmitoyl sphingomyelin (d18:1/16:0)	-0.38	0.0020	0.21	0.00029	0.0062
Fusobacterium	sphingomyelin (d18:1/21:0, d17:1/22:0, d16:1/23:0)*	-0.38	0.0020	0.21	0.00083	0.014
Fusobacterium	sphingomyelin (d18:2/23:0, d18:1/23:1, d17:1/24:1)*	-0.36	0.0020	0.21	0.000004	0.00022
Fusobacterium	palmitoyl dihydrosphingomyelin (d18:0/16:0)*	-0.36	0.0020	0.21	0.00020	0.0050
Fusobacterium	sphingomyelin (d18:0/20:0, d16:0/22:0)*	-0.36	0.0020	0.21	0.00003	0.0014
Turicibacter	trigonelline (N'-methylnicotinate)	-0.35	0.0788	0.62	0.00038	0.0077
Fusobacterium	sphingomyelin (d18:1/24:1, d18:2/24:0)*	-0.35	0.0020	0.21	0.00094	0.015
Fusobacterium	tricosanoyl sphingomyelin (d18:1/23:0)*	-0.33	0.0020	0.21	0.00002	0.00078
Fusobacterium	sphingomyelin (d18:2/24:1, d18:1/24:2)*	-0.33	0.0020	0.21	0.00080	0.014
Fusobacterium	sphingomyelin (d18:2/21:0, d16:2/23:0)*	-0.33	0.0020	0.21	0.00001	0.00060
Fusobacterium	sphingomyelin (d18:1/22:1,	-0.32	0.0020	0.21	0.0034	0.036

	d18:2/22:0, d16:1/24:1)*					
Fusobacterium	sphingomyelin (d18:1/22:2, d18:2/22:1, d16:1/24:2)*	-0.32	0.0020	0.21	0.0014	0.019
Fusobacterium	glycosyl-N-palmitoyl-sphingosine (d18:1/16:0)	-0.31	0.0020	0.21	0.0028	0.032
Ruminococcaceae_UCG-003	N-acetyl-2-aminooctanoate*	-0.31	0.0077	0.40	0.00002	0.00097
Dialister	tetrahydrocortisone glucuronide (5)	-0.30	0.0628	0.61	0.0023	0.028
Fusobacterium	sphingomyelin (d18:2/14:0, d18:1/14:1)*	-0.30	0.0020	0.21	0.0030	0.033

[a]Negative correlation indicates an inverse relationship between the metabolite and bacteria. Positive correlation indicates a relationship in the same direction between the metabolite and bacteria. We show only those with an absolute correlation ≥ ±0.3.
[b]This is the unadjusted p-value to select microbes for the correlation analysis.
[c]Benjamini-Hochberg adjusted p-value based on a two-sided correlation test.
[d]The exact p-value is 3.11e-13.
[e]The exact p-value is 0.0000009.

Supplementary Table 11. Multi-omics of third trimester samples between structural-zero genera and metabolites (N=88)

Genus	Chemical	Correlation[a]	Chemical p-value	Chemical BH-adjusted p-value[c]
Tyzzerella_4	2-hydroxyglutarate	0.44	0.00098	0.015
Peptoniphilus	2-hydroxyglutarate	0.39	0.00098	0.015
Fournierella	lithocholate sulfate (1)	0.36	0.0029	0.032
Rikenellaceae_RC9_gut_group	palmitoyl sphingomyelin (d18:1/16:0)	0.35	0.00029	0.0062
Fournierella	glycolithocholate sulfate*	0.33	0.001	0.015
Tyzzerella_4	glycosyl ceramide (d18:1/20:0, d16:1/22:0)*	-0.51	0.0005	0.0085
Peptoniphilus	glycosyl ceramide (d18:1/20:0, d16:1/22:0)*	-0.49	0.0005	0.0085
Peptoniphilus	hydroxypalmitoyl sphingomyelin (d18:1/16:0(OH))**	-0.49	<0.000001[b]	0.00006
Peptoniphilus	palmitoyl-sphingosine-phosphoethanolamine (d18:1/16:0)	-0.47	0.00003	0.0011
Tyzzerella_4	hydroxypalmitoyl sphingomyelin (d18:1/16:0(OH))**	-0.47	<0.000001[b]	0.00006
Tyzzerella_4	palmitoyl-sphingosine-phosphoethanolamine (d18:1/16:0)	-0.46	0.00003	0.0011
Cetobacterium	glycosyl-N-stearoyl-sphingosine (d18:1/18:0)	-0.44	0.00025	0.0055

Peptoniphilus	palmitoyl sphingomyelin (d18:1/16:0)	-0.43	0.00029	0.0062
Tyzzerella_4	palmitoyl sphingomyelin (d18:1/16:0)	-0.42	0.00029	0.0062
Murdochiella	hydroxypalmitoyl sphingomyelin (d18:1/16:0(OH))**	-0.41	<0.000001[b]	0.00006
Peptoniphilus	sphingomyelin (d18:1/24:1, d18:2/24:0)*	-0.41	0.00094	0.015
Tyzzerella_4	myristoyl dihydrosphingomyelin (d18:0/14:0)*	-0.40	0.0023	0.028
Tyzzerella_4	sphingomyelin (d18:1/24:1, d18:2/24:0)*	-0.40	0.00094	0.015
Tyzzerella_4	sphingomyelin (d18:2/23:1)*	-0.40	0.0017	0.022
Murdochiella	palmitoyl-sphingosine-phosphoethanolamine (d18:1/16:0)	-0.40	0.00003	0.0011
Murdochiella	glycosyl ceramide (d18:1/20:0, d16:1/22:0)*	-0.39	0.0005	0.009
Tyzzerella_4	sphingomyelin (d18:1/21:0, d17:1/22:0, d16:1/23:0)*	-0.39	0.00083	0.014
Peptoniphilus	myristoyl dihydrosphingomyelin (d18:0/14:0)*	-0.39	0.0023	0.028
Peptoniphilus	sphingomyelin (d18:2/23:1)*	-0.39	0.0017	0.022

Peptoniphilus	sphingomyelin (d18:1/21:0, d17:1/22:0, d16:1/23:0)*	-0.39	0.00083	0.014
Tyzzerella_4	palmitoyl dihydrosphingomyelin (d18:0/16:0)*	-0.39	0.0002	0.005
Tyzzerella_4	sphingomyelin (d18:2/23:0, d18:1/23:1, d17:1/24:1)*	-0.38	0.000004	0.00022
Peptoniphilus	palmitoyl dihydrosphingomyelin (d18:0/16:0)*	-0.38	0.0002	0.005
Tyzzerella_4	tricosanoyl sphingomyelin (d18:1/23:0)*	-0.38	0.00002	0.00078
Peptoniphilus	sphingomyelin (d18:2/23:0, d18:1/23:1, d17:1/24:1)*	-0.38	0.000004	0.00022
Peptoniphilus	sphingomyelin (d18:1/22:1, d18:2/22:0, d16:1/24:1)*	-0.38	0.0034	0.036
Peptoniphilus	sphingomyelin (d18:2/24:1, d18:1/24:2)*	-0.37	0.0008	0.014
Tyzzerella_4	sphingomyelin (d18:2/24:1, d18:1/24:2)*	-0.37	0.0008	0.014
Tyzzerella_4	sphingomyelin (d18:1/22:1, d18:2/22:0, d16:1/24:1)*	-0.37	0.0034	0.036

Tyzzerella_4	glycosyl-N-palmitoyl-sphingosine (d18:1/16:0)	-0.36	0.0028	0.032
Peptoniphilus	lactosyl-N-palmitoyl-sphingosine (d18:1/16:0)	-0.36	0.00023	0.0054
Peptoniphilus	tricosanoyl sphingomyelin (d18:1/23:0)*	-0.36	0.00002	0.00078
Peptoniphilus	glycosyl-N-palmitoyl-sphingosine (d18:1/16:0)	-0.35	0.0028	0.032
Peptoniphilus	sphingomyelin (d18:2/21:0, d16:2/23:0)*	-0.35	0.00001	0.0006
Tyzzerella_4	sphingomyelin (d18:0/20:0, d16:0/22:0)*	-0.35	0.00003	0.0014
Peptoniphilus	sphingomyelin (d18:1/22:2, d18:2/22:1, d16:1/24:2)*	-0.35	0.0014	0.019
Tyzzerella_4	lactosyl-N-palmitoyl-sphingosine (d18:1/16:0)	-0.35	0.00023	0.0054
Finegoldia	glycosyl-N-(2-hydroxynervonoyl)-sphingosine (d18:1/24:1(2OH))*	-0.35	0.000006	0.00031
Murdochiella	sphingomyelin (d18:2/21:0, d16:2/23:0)*	-0.35	0.00001	0.0006
Tyzzerella_4	1-(1-enyl-palmitoyl)-2-palmitoyl-GPC (P-16:0/16:0)*	-0.34	0.0029	0.033

Peptoniphilus	sphingomyelin (d18:0/20:0, d16:0/22:0)*	-0.34	0.00003	0.0014
Tyzzerella_4	lignoceroyl sphingomyelin (d18:1/24:0)	-0.34	0.0018	0.023
Tyzzerella_4	sphingomyelin (d18:2/21:0, d16:2/23:0)*	-0.34	0.00003	0.0006
Fournierella	3-aminoisobutyrate	-0.33	0.0002	0.005
Murdochiella	sphingomyelin (d18:1/22:2, d18:2/22:1, d16:1/24:2)*	-0.33	0.0014	0.019
Peptoniphilus	1-(1-enyl-palmitoyl)-2-palmitoyl-GPC (P-16:0/16:0)*	-0.33	0.0029	0.033
Murdochiella	palmitoyl sphingomyelin (d18:1/16:0)	-0.33	0.00029	0.0062
Tyzzerella_4	2-methylbutyrylcarnitine (C5)	-0.33	0.0013	0.019
Murdochiella	lactosyl-N-palmitoyl-sphingosine (d18:1/16:0)	-0.33	0.00023	0.0054
Peptoniphilus	2-methylbutyrylcarnitine (C5)	-0.33	0.0013	0.019
Tyzzerella_4	sphingomyelin (d18:1/22:2, d18:2/22:1, d16:1/24:2)*	-0.33	0.0014	0.019
Tyzzerella_4	sphingomyelin (d18:2/14:0, d18:1/14:1)*	-0.33	0.003	0.033

Murdochiella	sphingomyelin (d18:2/24:1, d18:1/24:2)*	-0.32	0.0008	0.014
Murdochiella	glycosyl-N-(2-hydroxynervonoyl)-sphingosine (d18:1/24:1(2OH))*	-0.32	0.000006	0.00031
Murdochiella	sphingomyelin (d18:1/24:1, d18:2/24:0)*	-0.32	0.00094	0.015
Peptoniphilus	lignoceroyl sphingomyelin (d18:1/24:0)	-0.32	0.0018	0.023
Peptoniphilus	sphingomyelin (d18:2/14:0, d18:1/14:1)*	-0.32	0.003	0.033
Murdochiella	glycosyl ceramide (d18:2/24:1, d18:1/24:2)*	-0.32	0.00007	0.0021
Peptoniphilus	glycosyl-N-stearoyl-sphingosine (d18:1/18:0)	-0.32	0.00025	0.0055
Peptoniphilus	glycosyl ceramide (d18:2/24:1, d18:1/24:2)*	-0.32	0.00007	0.0021
Rikenellaceae_RC9_gut_group	cis-4-decenoylcarnitine (C10:1)	-0.31	0.0018	0.022
Tyzzerella_4	glycosyl-N-stearoyl-sphingosine (d18:1/18:0)	-0.31	0.00025	0.0055
Murdochiella	sphingomyelin (d18:2/23:1)*	-0.31	0.0017	0.022
Eggerthella	glycocholenate sulfate*	-0.31	0.0000035	0.00022

[a]Negative correlation indicates an inverse relationship between the metabolite and bacteria. Positive correlation indicates a relationship in the same direction between the metabolite and bacteria. We show only those with an absolute correlation $\geq \pm 0.3$.
[b]The exact p-value is 8.6e-07.
[c]Benjamini-Hochberg adjusted p-value based on a two-sided correlation test.

Supplementary Figure 2. Multi-omics at the third trimester by HIV status between structural zero genera and metabolites (N=88)

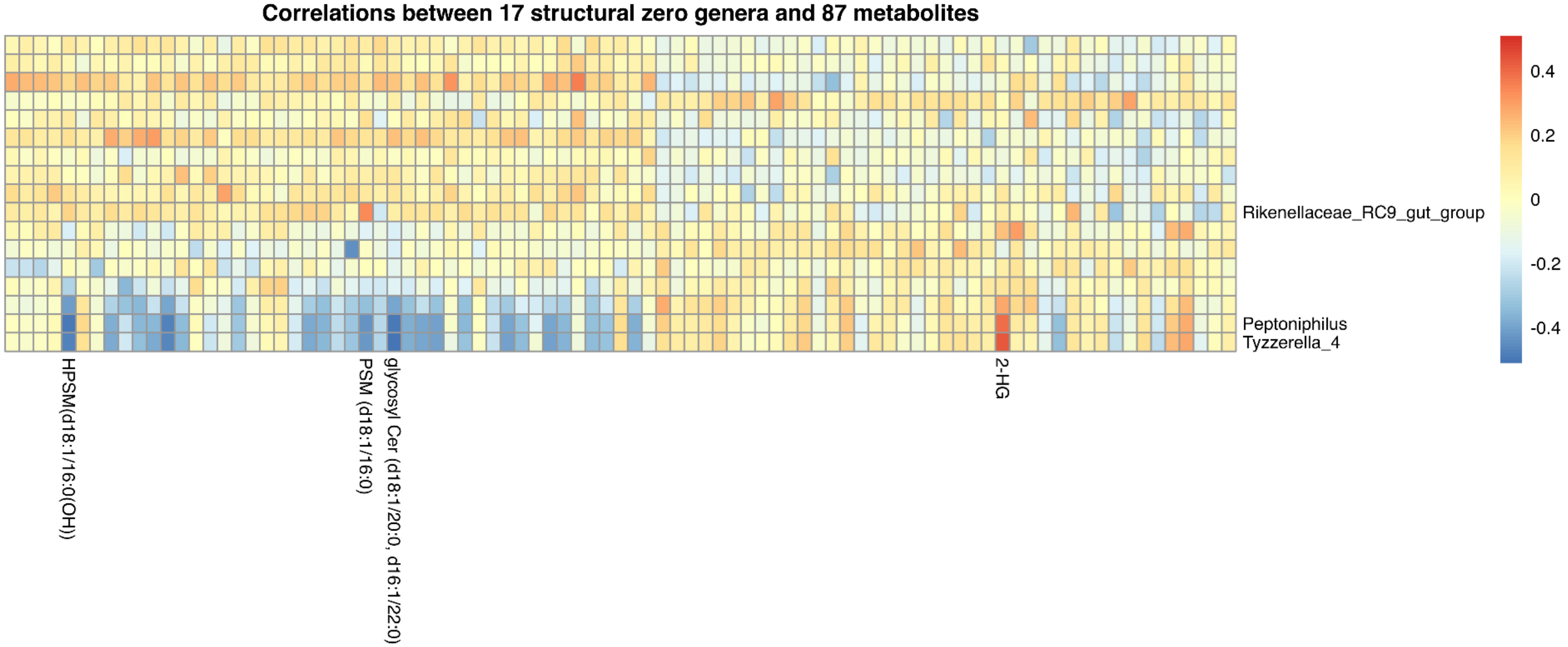

Supplementary Figure 2 Legend. The heat map shows the correlation between 17 differentially present/absent structural zero genera from our Analysis of Compositions of Microbiomes with Bias Correction (ANCOM-BC) analysis and 87 significant metabolites from our metabolomics analysis in a multi-omics analysis of 88 third trimester samples paired with metabolomics and microbiota data using a two-sided correlation test. Those in blue have a high negative correlation and those in red have a high positive correlation. Metabolite and microbe associations with high negative or positive correlations are labeled. Due to the large number of metabolites and microbes, we only label select bacteria and metabolites with high positive or negative correlations. The specific correlations with an absolute correlation ≥ ±0.3 between microbes and metabolites are shown in Supplementary Table 11. Abbreviation: HPSM(d18:1/16:0(OH)) for hydroxypalmitoyl sphingomyelin (d18:1/16:0(OH)), PSM (d18:1/16:0) for palmitoyl sphingomyelin (d18:1/16:0), glycosyl Cer (d18:1/20:0, d16:1/22:0) for glycosyl ceramide (d18:1/20:0, d16:1/22:0), and 2-HG for 2-hydroxyglurate.

Supplementary Figure 3. Gut microbial α-diversity at 6 months post-partum by HIV status (N=182)

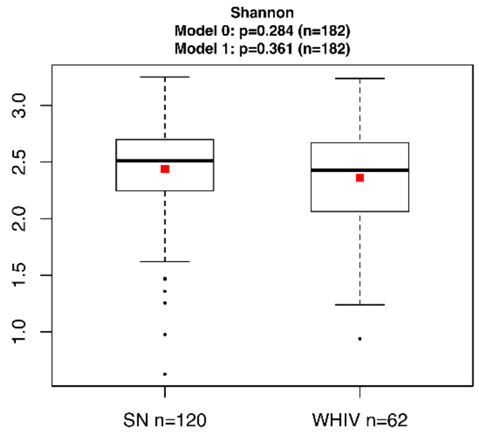

Supplementary Figure 3 Legend. Comparison of α-diversities between pregnant WHIV and SN at 6 months postpartum. Univariable and multivariable regression models were used to calculate p-values. The 2 models were tested using the Shannon index to examine α-diversity. Model 0 used a t-test with no adjustments. Model 1 used regression models and adjusted for age, mid-upper arm circumference (MUAC), and education. WHIV indicates women with HIV. SN indicates women seronegative for HIV. The thick black line represents the median, the red dot is the mean, the box shows the interquartile range (IQR) with the bottom line as the 25th percentile and top line as the 75th percentile, the lines extending from the box are the whiskers (1.5 x IQR), and dots after the whiskers are outliers.

Supplementary Figure 4. Gut microbial α-diversity at 6 months of infant age by HIV exposure status (N=177)

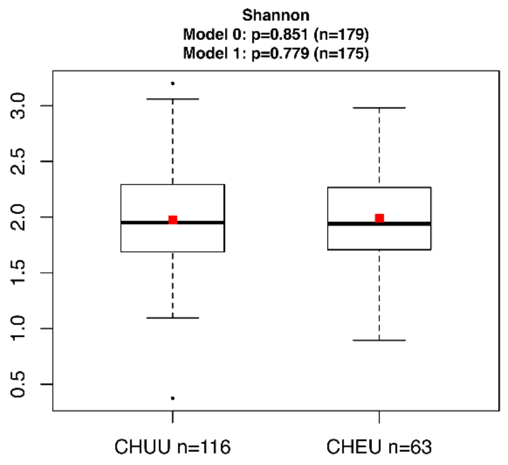

Supplementary Figure 4 Legend. Comparison of α-diversities between children who are HIV-exposed uninfected (CHEU) and children who are HIV-unexposed uninfected (CHUU) at 6 months of age. T-tests and multivariable regression models were used to calculate p-values. The 2 models were tested using the Shannon index to examine α-diversity. Model 0 used a t-test with with no adjustments. Model 1 used a regression model and adjusted for maternal age, infant BMI z-score at 6 months, and maternal education. CHEU indicates exposed to HIV through mother. CHUU indicates not exposed to HIV through mother. The thick black line represents the median, the red dot is the mean, the box shows the interquartile range (IQR) with the bottom line as the 25th percentile and top line as the 75th percentile, the lines extending from the box are the whiskers (1.5 x IQR), and dots after the whiskers are outliers.

Supplementary Figure 5. Gut microbial α-diversity at 6 months of infant age who are exclusively breastfed (N=60) and who are not exclusively breastfeeding (N=116) by HIV exposure status

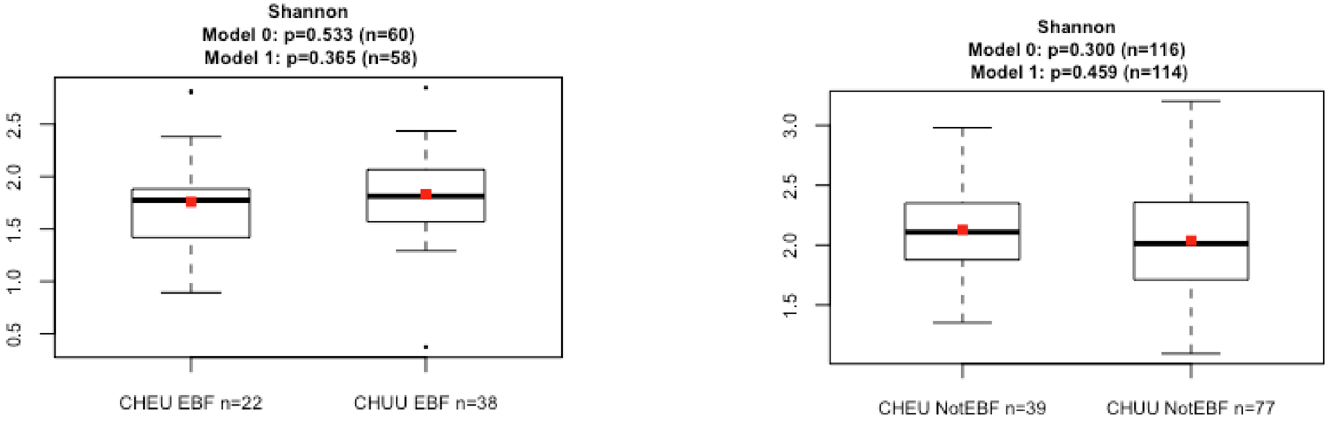

a. **b.**

Supplementary Figure 5 Legend. Comparison of α-diversities between children who are HIV-exposed uninfected (CHEU) and children who are HIV-unexposed uninfected (CHUU) at 6 months of age who are: **a.** exclusively breastfed and **b.** not exclusively breastfed. T-tests and multivariable regression models were used to calculate p-values. The 2 models were tested using the Shannon index to examine α-diversity. Model 0 used T-tests with no adjustments. Model 1 adjusted for maternal age, infant BMI z-score at 6 months, and maternal education. CHEU indicates exposed to HIV through mother. CHUU indicates not exposed to HIV through mother. EBF indicates exclusive breastfeeding. NotEBF indicates not exclusive breastfeeding. The thick black line represents the median, the red dot is the mean, the box shows the interquartile range (IQR) with the bottom line as the 25th percentile and top line as the 75th percentile, the lines extending from the box are the whiskers (1.5 x IQR), and dots after the whiskers are outliers.

Supplementary Figure 6. Differences in the abundance of taxa at the genus level at 6 months of infant age who are not exclusively breastfed by HIV exposure status (N=116)

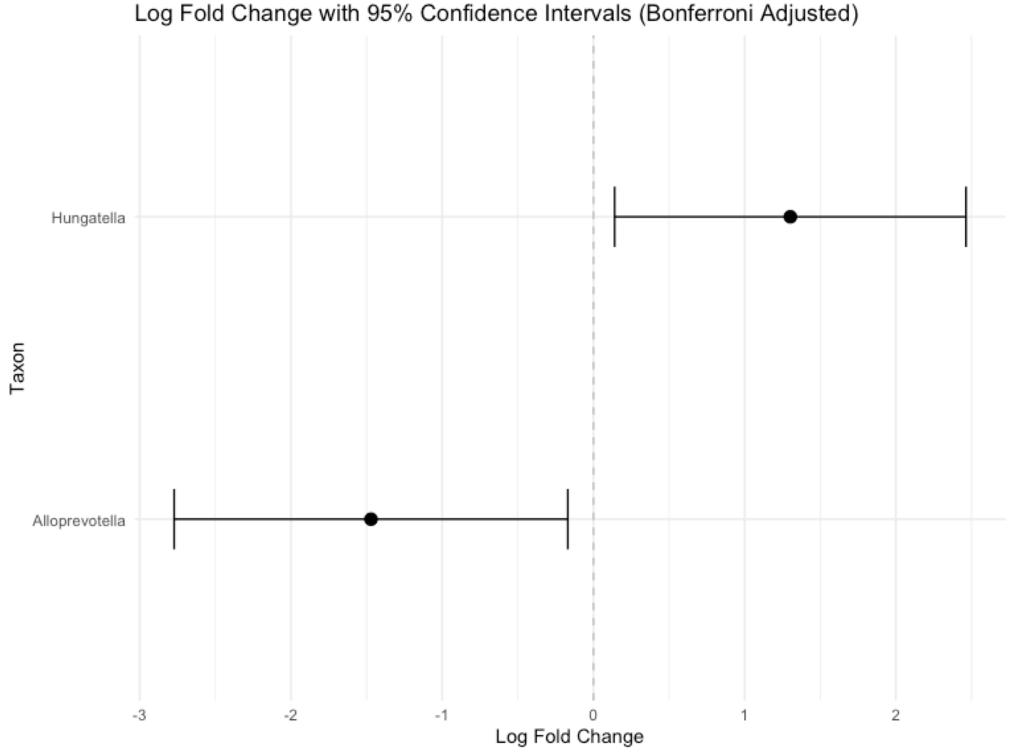

Supplementary Figure 6 Legend. The log fold change with 95% confidence intervals plot represents the gut microbiota profiles of infants 6 months of age who are not exclusively breastfed by HIV exposure status using the Analysis of Compositions of Microbiomes with Bias Correction (ANCOM-BC) method. It shows the Bonferroni adjusted log fold change value (black dot) on the x-axis with the bars representing the lower and upper bound of the 95% confidence interval. As there were no bacteria that were significant after FDR adjustment, this figure shows taxa that had raw p-values <0.05 for children who are HIV-exposed uninfected (CHEU) at 6 months of age who are exclusively breastfed for Model 1, adjusted for maternal age, infant BMI z-score at 6 months, and maternal education. The log fold-change (LFC) represents the log change in corrected absolute abundance of bacteria in Model 1 (e.g. higher or lower) of

CHEUs who are exclusively breastfed as compared to children who are HIV-unexposed uninfected (CHUU) who are exclusively breastfed. Error bars indicate 95% confidence intervals (CI) of the LFC estimates. LFC and confidence interval bars to the left of zero represents a lower abundance of bacteria in the CHEU group who are not exclusively breastfed compared to the CHUU group and LFC and confidence interval bars to the right of zero represent a higher abundance of bacteria in the CHEU group who are not exclusively breastfed compared to the CHUU group.

Supplementary Figure 7. Gut microbial α-diversity of non-pregnant women by HIV status (N=173)

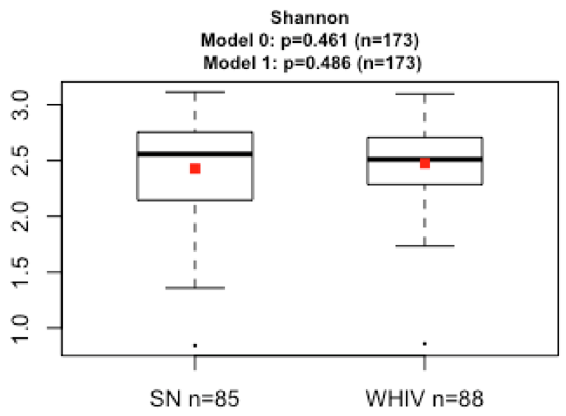

Supplementary Figure 7 Legend. Comparison of α-diversities between non-pregnant WHIV and SN. T-tests and multivariable regression models were used to calculate p-values. The 2 models were tested using the Shannon index to examine α-diversity. Model 0 used T-tests with no adjustments. Model 1 adjusted for age, MUAC, and education. WHIV indicates women with HIV. SN indicates women seronegative for HIV. The thick black line represents the median, the red dot is the mean, the box shows the interquartile range (IQR) with the bottom line as the 25th percentile and top line as the 75th percentile, the lines extending from the box are the whiskers (1.5 x IQR), and dots after the whiskers are outliers.

Supplementary Figure 8. Differential abundance of taxa at the genus level of non-pregnant women by HIV status (N=173)

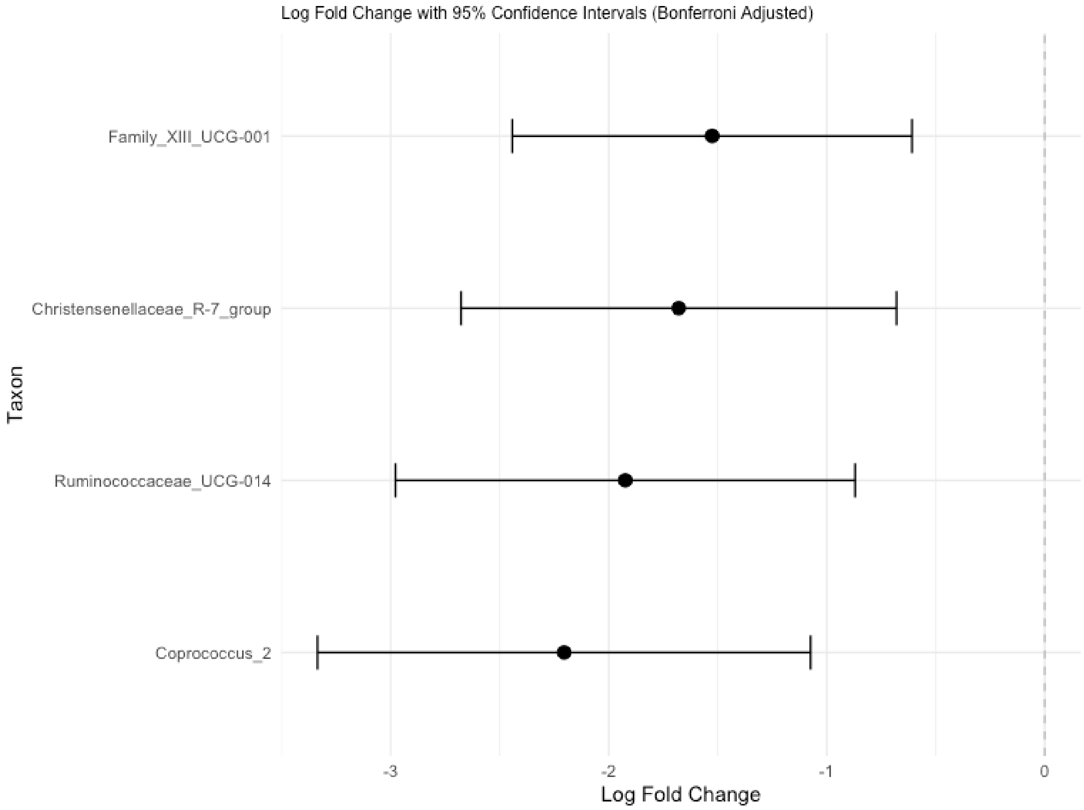

Supplementary Figure 8 Legend. The log fold change with 95% confidence intervals plot represents the gut microbiota profiles of non-pregnant women by HIV status using the Analysis of Compositions of Microbiomes with Bias Correction (ANCOM-BC) method. It shows the Bonferroni adjusted log fold change value (black dot) on the x-axis with the bars representing the lower and upper bound of the 95% confidence interval. This is shown for each statistically significant taxa (y-axis) from the Benjamini-Hochberg adjusted p-values (threshold of <0.05) for non-pregnant women with HIV for Model 1, adjusted for age, MUAC, and education. The log fold-change (LFC) represents the log change in corrected absolute abundance of bacteria in Model 1 (e.g. higher or lower) of non-pregnant WHIV as compared to SN. Error bars indicate 95% confidence intervals (CI) of the LFC estimates. LFC and confidence interval bars to the left of zero represent a lower abundance of bacteria in the non-pregnant WHIV group compared to the SN group and LFC and

confidence interval bars to the right of zero represent a higher abundance of bacteria in the non-pregnant WHIV group compared to the SN group.

<u>Supplementary Table 12.</u> Differential abundance of taxa of non-pregnant women by HIV status (N=173)

	Model 0		Model 1[a]	
Taxon[e]	LFC[c] [CI][d]	P-value[b]	LFC [CI]	P-value
Coprococcus_2	-1.9 [-2.98, -0.81]	*0.0414*[e]	-2.21 [-3.34, -1.07]	*0.0197*
Ruminococcaceae_UCG-014	-1.84 [-2.82, -0.858]	*0.0334*	-1.92 [-2.98, -0.87]	*0.0242*
Christensenallaceae_R-7_group	-1.38 [-2.35, -0.408]	0.126	-1.68 [-2.68, -0.68]	*0.0356*
Family_XIII_UCG-001	-1.26 [-2.14, -0.375]	0.126	-1.53 [-2.44, -0.609]	*0.0356*

[a]Multivariable model 1 adjusted for age, middle upper arm circumference (MUAC), and education.
[b]Benjamini-Hochberg adjusted p-values
[c]The Bonferroni adjusted log fold change (LFC) represents the logarithmic change in corrected absolute abundance of bacteria in the specified model (e.g. higher or lower) in non-pregnant WHIV and SN. If the estimate is positive, higher in non-pregnant WHIV and if negative, lower in non-pregnant WHIV.
[d]The Bonferroni adjusted 95% confidence interval indicates a range of values, upper and lower bound, within which the true LFC lies for each taxa.
[e]Italicized p-values indicate bacteria that are significant for each model.
[f]The log fold change of *Coprococcus_2* in adjusted model 1 is -2.21 lower in non-pregnant WHIV compared to those SN, with a 95% confidence interval of [-3.34, -1.07], indicating a statistically significant decrease in abundance based on the p-value of 0.0197 which is < 0.05. ANCOM-BC analysis methods were used for this which is a two-sided test.

1. Caporaso JG*, et al.* QIIME allows analysis of high-throughput community sequencing data. *Nat Methods* **7**, 335–336 (2010).

2. Schmieder R, Lim YW, Rohwer F, Edwards R. TagCleaner: Identification and removal of tag sequences from genomic and metagenomic datasets. *BMC Bioinformatics* **11**, 341 (2010).

3. Callahan BJ, McMurdie PJ, Rosen MJ, Han AW, Johnson AJ, Holmes SP. DADA2: High-resolution sample inference from Illumina amplicon data. *Nat Methods* **13**, 581–583 (2016).

4. Jayanama K*, et al.* Association between gut microbiota and prediabetes in people living with HIV. *Current Research in Microbial Sciences* **3**, 100143 (2022).

5. Dong R*, et al.* Gut Microbiota and Fecal Metabolites Associated With Neurocognitive Impairment in HIV-Infected Population. *Frontiers in Cellular and Infection Microbiology* **Volume 11 - 2021**, (2021).
