# Supplementary material for: Impact of maternal HIV infection on the gut microbiome and metabolome of mothers and infants: the PRACHITi cohort in Pune, India
Source: Nat Commun. 2026 Feb 23;17:3097. doi: 10.1038/s41467-026-69912-0 (PMC13039106; doi:10.1038/s41467-026-69912-0)
Supplement: Supplementary file 2 — Reporting Summary [file 41467_2026_69912_MOESM2_ESM.pdf]

nature portfolio

Corresponding author(s): Rupak Shivakoti

Last updated by author(s): Jan 29, 2026

Reporting Summary

Nature Portfolio wishes to improve the reproducibility of the work that we publish. This form provides structure for consistency and transparency in reporting. For further information on Nature Portfolio policies, see our Editorial Policies and the Editorial Policy Checklist.

Statistics

For all statistical analyses, confirm that the following items are present in the figure legend, table legend, main text, or Methods section.

n/a	Confirmed	
☐	☒	The exact sample size (*n*) for each experimental group/condition, given as a discrete number and unit of measurement
☐	☒	A statement on whether measurements were taken from distinct samples or whether the same sample was measured repeatedly
☐	☒	The statistical test(s) used AND whether they are one- or two-sided<br>*Only common tests should be described solely by name; describe more complex techniques in the Methods section.*
☐	☒	A description of all covariates tested
☐	☒	A description of any assumptions or corrections, such as tests of normality and adjustment for multiple comparisons
☐	☒	A full description of the statistical parameters including central tendency (e.g. means) or other basic estimates (e.g. regression coefficient) AND variation (e.g. standard deviation) or associated estimates of uncertainty (e.g. confidence intervals)
☒	☐	For null hypothesis testing, the test statistic (e.g. *F*, *t*, *r*) with confidence intervals, effect sizes, degrees of freedom and *P* value noted<br>*Give P values as exact values whenever suitable.*
☒	☐	For Bayesian analysis, information on the choice of priors and Markov chain Monte Carlo settings
☐	☒	For hierarchical and complex designs, identification of the appropriate level for tests and full reporting of outcomes
☒	☐	Estimates of effect sizes (e.g. Cohen's *d*, Pearson's *r*), indicating how they were calculated

Our web collection on statistics for biologists contains articles on many of the points above.

Software and code

Policy information about availability of computer code

Data collection	No software was used for data collection.
Data analysis	Amplicon sequencing of the V3-V4 regions of the 16s rRNA gene was conducted through a two-step PCR process. Sequences were de-multiplexed using QIIME scripts, primer sequences removed with TagCleaner version 0.16 and DADA2 workflow was used for data processing. Taxonomic assignments were made using the RDP Classifier and SILVA v138 database. Metabolon's proprietary software was used for metabolite peak detection, compound identification, and quality control. R version 4.5.1 was used for all statistical analyses. Alpha diversity boxplots were created using the phyloseq and microbiome package. PERMANOVA for beta diversity was run through the vegan package and Principal Coordinates Analysis plots (PCoA) were generated. Differential abundance analyses were conducted using the ANCOMBC package, and linear mixed models were performed using the lme function from the nlme package. Code and data files are in GitHub (https://github.com/tianwang-wow/Pregnancy_microbiome_in_HIV).

For manuscripts utilizing custom algorithms or software that are central to the research but not yet described in published literature, software must be made available to editors and reviewers. We strongly encourage code deposition in a community repository (e.g. GitHub). See the Nature Portfolio guidelines for submitting code & software for further information.

Data

Policy information about availability of data

All manuscripts must include a data availability statement. This statement should provide the following information, where applicable:

- Accession codes, unique identifiers, or web links for publicly available datasets
- A description of any restrictions on data availability
- For clinical datasets or third party data, please ensure that the statement adheres to our policy

The raw sequencing microbiome data and associated metadata have been deposited to the sequence read archive repository with the BioProject Number PRJNA1224750 (http://www.ncbi.nlm.nih.gov/bioproject/1224750).

Research involving human participants, their data, or biological material

Policy information about studies with human participants or human data. See also policy information about sex, gender (identity/presentation), and sexual orientation and race, ethnicity and racism.

Reporting on sex and gender	The findings are applied to only one sex: female. We mentioned in the title that this is among maternal populations and in the abstract that it is among pregnant women with and without HIV. Women enrolled were confirmed pregnant through an early pregnancy ultrasonography and their sex is self-reported when enrolled. Infant sex was not considered in the study design but to note there were 80 male infants and 93 female infants and 4 missing gender out of 177 infants in our analysis.
Reporting on race, ethnicity, or other socially relevant groupings	All participants identified as Asian and all participants did not identify as Hispanic or Latino. Since race and ethnicity was homogeneous for all participants. We used education to account for socioeconomic status. It was categorized as none to high school and post high school to postgraduate.
Population characteristics	The population consisted of 207 pregnant women in their third trimester with a median age of 23 and IQR of 21-27.here were 75 pregnant women with HIV (36%) and 132 seronegative for HIV (64%). All HIV+ participants were on some type of ART regimen with 73% on TDF/3TC/EFV. Of the total population, 89% had none to high school educational experience and 11% had post high school to postgraduate experience. There were more WHIV with undernutrition (36%) than those seronegative for HIV (28%).
Recruitment	Participants were recruited through the antenatal clinic at Sassoon Hospital in Pune, India who were pregnant women between the ages of 18 and 40 and between 13 and 34 weeks of gestation as confirmed by early pregnancy ultrasonography. Exclusion criteria included women with active TB, severe anemia, ongoing use of antibiotics for more than 14 days, autoimmune or immunosuppressive diseases, or current used of immunosuppressive medication.
Ethics oversight	Approval for the study was obtained from the institutional ethics review boards of BJGMC, the Johns Hopkins University, Columbia University and Weill Cornell Medicine, as well as the Health Ministry Screening Committee of the Indian Council Medical Research, India. All guidelines required by the US Department of Health and Human Services for Human experimentation were followed. We received written informed consent from all participants. The reporting guideline for observational studies: Strengthening the Reporting of Observational Studies in Epidemiology (STROBE) was followed.

Note that full information on the approval of the study protocol must also be provided in the manuscript.

Field-specific reporting

Please select the one below that is the best fit for your research. If you are not sure, read the appropriate sections before making your selection.

[x] Life sciences [ ] Behavioural & social sciences [ ] Ecological, evolutionary & environmental sciences

For a reference copy of the document with all sections, see nature.com/documents/nr-reporting-summary-flat.pdf

Life sciences study design

All studies must disclose on these points even when the disclosure is negative.

Sample size	Overall, 245 pregnant women, stratified by HIV status, were enrolled and followed through 1 year postpartum. The sample size for PRACHITi was determined based on the primary objectives related to immune responses to TB antigens for pregnant women. for this analysis, we used data from the same women followed longitudinally and utilized a complete-case approach.
Data exclusions	No data was excluded from the analyses.
Replication	This was an observational cohort study examining association between HIV status and maternal-infant gut microbiome, direct experimental replication was not applicable.
Randomization	This was an observational study without experimental groups, so randomization was not applicable. The two groups in our study were HIV+ and HIV- and were determined by HIV testing. Potential confounding variables (age, education, anthropometrics) were controlled for in statistical analyses.
Blinding	This was an observational study without intervention groups, so blinding was not applicable.

Reporting for specific materials, systems and methods

We require information from authors about some types of materials, experimental systems and methods used in many studies. Here, indicate whether each material, system or method listed is relevant to your study. If you are not sure if a list item applies to your research, read the appropriate section before selecting a response.

Materials & experimental systems

n/a	Involved in the study
☒	☐ Antibodies
☒	☐ Eukaryotic cell lines
☒	☐ Palaeontology and archaeology
☒	☐ Animals and other organisms
☐	☒ Clinical data
☒	☐ Dual use research of concern
☒	☐ Plants

Methods

n/a	Involved in the study
☒	☐ ChIP-seq
☒	☐ Flow cytometry
☒	☐ MRI-based neuroimaging

Clinical data

Policy information about clinical studies

All manuscripts should comply with the ICMJE guidelines for publication of clinical research and a completed CONSORT checklist must be included with all submissions.

Clinical trial registration	N/A - this was not a randomized trial. It was an observational cohort study.
Study protocol	Study protocol is available from the corresponding author upon reasonable request.
Data collection	Data collection occurred in Pune, India at Byramjee Jeejeebhoy Government Medical College (BJGMC), a tertiary care hospital serving low-income populations and acting as a referral center for HIV care. The study time period was from June 27, 2016 and ended on December 9, 2019.
Outcomes	Outcomes were maternal and infant microbiomes analysed by 16s rRNA amplicon sequencing.

Plants

Seed stocks	N/A
Novel plant genotypes	N/A
Authentication	N/A
